# *QuickStats:* Number of Deaths from Hornet, Wasp, and Bee Stings,* Among Males and Females — National Vital Statistics System, United States,^†^ 2000–2017

**DOI:** 10.15585/mmwr.mm6829a5

**Published:** 2019-07-26

**Figure:**
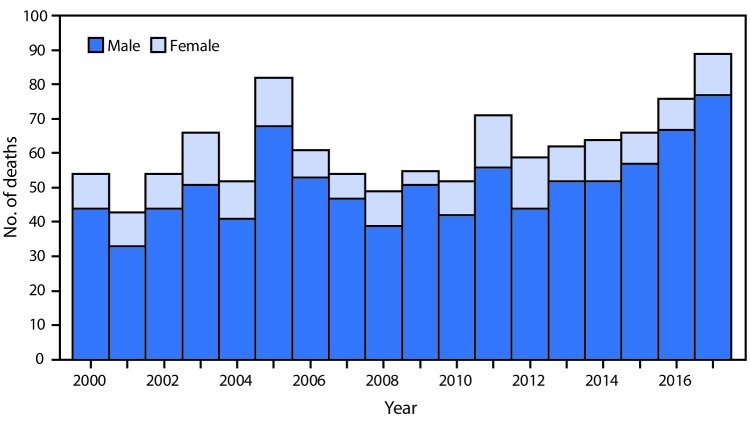
During 2000–2017, a total of 1,109 deaths from hornet, wasp, and bee stings occurred, for an annual average of 62 deaths. Deaths ranged from a low of 43 in 2001 to a high of 89 in 2017. Approximately 80% of the deaths were among males.

**Source:** National Vital Statistics System. Underlying cause of death data, 1999–2017. https://wonder.cdc.gov/ucd-icd10.html.

